# Long-Term Statin Administration Does Not Affect Warfarin Time in Therapeutic Range in Australia or Singapore

**DOI:** 10.3390/jcm7050097

**Published:** 2018-05-01

**Authors:** Nijole Bernaitis, Chi Keong Ching, Siew Chong Teo, Tony Badrick, Andrew K. Davey, Julia Crilly, Shailendra Anoopkumar-Dukie

**Affiliations:** 1 Quality Use of Medicines Network, and Menzies Health Institute, School of Pharmacy & Pharmacology, Griffith University, Nathan, QLD 4215, Australia; n.bernaitis@griffith.edu.au (N.B.); a.davey@griffith.edu.au (A.K.D.); 2 Cardiology Department, National Heart Centre, Singapore 169609, Singapore; ching.chi.keong@nhcs.com.sg; 3 Pharmacy Department, National Heart Centre, Singapore 169609, Singapore; teo.siew.chong@nhcs.com.sg; 4 Royal College of Pathologists of Australasia Quality Assurance Programs, St Leonards, NSW 2065, Australia; Tony.Badrick@rcpaqap.com.au; 5 Department of Emergency Medicine Gold Coast Hospital and Health Service, Griffith University, Nathan, QLD 4215, Australia; Julia.Crilly@health.qld.gov.au

**Keywords:** warfarin: statins, HMG-CoA reductase inhibitors

## Abstract

Background: Warfarin requires ongoing monitoring of the International Normalised Ratio (INR). This is because numerous factors influence the response, including drug interactions with commonly-prescribed medications, such as statins. The administration of statins with warfarin may change INR; however, there is limited information regarding the effects on warfarin control as measured by time in therapeutic range (TTR). Statins may also alter bleeds with warfarin, but there are conflicting reports demonstrating both increased and decreased bleeds, and limited data on diverse ethnic populations. Therefore, the aim of this study was to determine the effect of statin administration on warfarin control and bleeds in patients in Australia and Singapore. Methods: Retrospective data were collected for patients on warfarin between January and June 2014 in Australia and Singapore. Patient data were used to calculate TTR and bleed events. Concurrent statin therapy was assessed and comparisons of TTR and bleed incidence were made across patient subgroups. Results: Warfarin control in Australia and Singapore was not significantly affected by statins, as measured by TTR (83% and 58%, respectively), frequency of testing, and warfarin doses. In Australia, statin use did not significantly affect bleeds, whilst in Singapore the bleed incidence was significantly lower for patients on statins. Conclusions: Chronic concurrent administration of statins with warfarin does not adversely affect warfarin TTR in Australia or Singapore. In Singapore, patients on statins, compared to no statins, had a lower bleed incidence and this requires further investigation, especially given the potential genetic influences of ethnicity on both statin and warfarin metabolism.

## 1. Introduction

Warfarin is a widely-prescribed anticoagulant used for the prevention of stroke in patients with atrial fibrillation (AF) [1]. Close monitoring of warfarin treatment is necessary due to the variations in individual responses to dosing and with the efficacy and safety of warfarin being dependent on maintaining levels within the therapeutic range [2]. Achieving the target International Normalised Ratio (INR) range is a measure of successful management of warfarin, with standards suggesting greater than 70% time in the therapeutic range (TTR) as being best [3]. Maintaining warfarin within a narrow therapeutic index is clinically challenging due to a number of influencing patient factors, including drug–drug interactions [4]. Drug interactions with warfarin may be pharmacokinetic, involving cytochrome p450 isoenzymes, or pharmacodynamics, resulting in increased bleeding, the most common adverse reaction to warfarin [5].

Drug interactions with warfarin represent a common cause of bleeding and over-anticoagulation, particularly in the elderly, with multiple co-morbidities and medications [6]. Wittkowsky et al. [7] found that almost 82% of patients receiving long-term warfarin were co-prescribed at least one potentially interacting drug. Furthermore, Rikala et al. [8] found that over 90% of warfarin users were co-prescribed cardiovascular drugs and over a third were prescribed a statin, i.e., simvastatin. The concurrent use of statins (3-hydroxy-3-methyl-glutaryl-CoA reductase inhibitors) and warfarin is, therefore, common, but the safety of the drug interactions requires further exploration [9]. The metabolism of the statins involves cytochrome p450 (CYP) 3A4 for simvastatin, atorvastatin, and lovastatin, CYP2C9 for fluvastatin and rosuvastatin, whilst pravastatin is not markedly metabolised by CYP [10]. Both CYP3A4 and 2C9 are involved in warfarin metabolism, so the co-administration of statins, which utilise these CYP pathways, can increase INR and increase the risk of bleeding [11]. However, there have been conflicting reports regarding the outcomes of the interactions of warfarin and statins, particularly regarding bleed risk. Schelleman et al. [12] found an increased risk of gastrointestinal bleeding with statins inhibiting CYP3A4, i.e., simvastatin and atorvastatin, but no change with fluvastatin metabolised via CYP2C9. Similar to this, Shin et al. [13] found that the incidence of gastrointestinal bleed risk was highest with rosuvastatin, followed by atorvastatin and simvastatin, while pravastatin tended to reduce the risk. Interestingly, despite the different bleed rates for the individual statins, these authors found no difference in TTR between the statin groups during the study period [13]. Further to this, Douketis et al. [14] associated long-term statin use with a decreased risk of warfarin bleeding; however, they did not include an anticoagulation control in the analysis or assess the risks of individual statins. The conflicting reports regarding the effects of statins on warfarin suggest further investigation is required into the associated risks with this drug combination. In addition, the influence of ethnicity on the outcomes of warfarin and statins requires further investigation. This is because the prescription of statins may be influenced by their potency, pharmacokinetics and the toxicity of the statins, particularly in Asian compared to Caucasian populations [15]. Therefore, the aim of this study was to determine the effects of statin administration on warfarin control and bleed risk in large cohorts of patients with AF, in both Australia and Singapore.

## 2. Methods

Ethics approval was obtained from Griffith University (PHM/09/14/HREC and PHM/08/15/HREC) and SingHealth Centralised Institutional Review Board (CIRB 2015/2435). A retrospective data analysis was conducted for patients receiving warfarin for non-valvular AF for the period of January 2014 to June 2014. Two groups were investigated, namely, patients enrolled in a warfarin care program at Sullivan Nicolaides Pathology, Queensland, Australia, and patients managed at the outpatient warfarin clinic at The National Heart Centre Singapore. The data collected included patients’ age, gender, co-morbidities, concurrent medications, warfarin doses, INR test dates and their results. The HASBLED (Hypertension, Abnormal renal/liver function, Stroke, Bleeding history, Labile International Normalised Ratio or INR, Elderly > 65 years, Drugs/alcohol concomitantly) score was calculated to assess bleed risk. Adverse events were also recorded, including self-reported bleeds (e.g., nose bleeds, rectal bleeding), and major bleeds or stroke requiring hospital admissions. Patient data were screened to identify those who were concurrently prescribed a statin during the study period, and divided into groups of statin users and no-statin users. Patients taking statins were further divided according to the individual statin co-administered with warfarin.

TTR was calculated using Rosendaal’s linear interpolation algorithm [16] with software downloaded from INR Pro©. Exclusions for analysis included patients for whom TTR was unable to be calculated (i.e., less than 2 tests), and a time of treatment of fewer than 30 days. Mean data were used for analysis and comparison between groups. Events were categorised into minor or major bleeds, stroke, and death, and calculated as an incidence per patient for each group for comparison purposes.

Patient characteristics were reported as number and percentage for categorical data, and mean ± standard deviation for continuous data. Statistical analysis was performed using GraphPad Instat Version 3 with comparisons made using ordinary analysis of variance through non-parametric methods, including Dunn’s multiple comparisons test and Kruskal-Wallis test. Event incidence was compared using two-sided Fisher’s exact test. Significance was defined as * or ^#^ *p* < 0.05, ** or ^##^ *p* < 0.01, *** or ^###^ *p* < 0.001, and **** or ^####^ *p* < 0.0001.

## 3. Results

A total of 4366 patients with non-valvular AF were included in the study, with 3196 patients in Australia and 1170 in Singapore. This was following exclusions for insufficient data to calculate TTR for 48 patients in Australia and 260 patients in Singapore. The mean age of patients was 77.2 ± 9.1 years in Australia and 69.7 ± 10.0 years in Singapore, which was statistically different (*p* < 0.0001) (Table 1). The majority of patients were male (52.2% in Australia and 60.3% in Singapore) and the median (IQR) HASBLED score was 1 (1–2) in both Australia and Singapore despite differences in the occurrence of co-morbidities. At both sites, the majority of patients were taking statins (57.3% in Australia and 73.4% in Singapore) and the majority of patients were prescribed either atorvastatin (53.4% in Australia, 21.9% in Singapore) or simvastatin (19.3% in Australia, 68.5% in Singapore). There was no significant difference in the mean age of patients taking statins and not taking statins (76.7 ± 8.6 vs. 77.8 ± 9.7 years in Australia and 70.1 ± 9.6 vs. 68.61 ± 1.0 years in Singapore).

The mean TTR was 82.4 ± 15.6% in Australia and 57.6 ± 34.2% in Singapore, which was statistically different (*p* < 0.0001) (Table 2). At the individual sites, no significant difference was found in the mean TTR, INR testing frequency, warfarin doses, or frequency of dose changes for patients with or without statin therapy. The mean TTR and frequency of testing did not differ significantly, regardless of the statin prescribed in either Australia or Singapore.

No statistical differences were found in bleed incidences between Australia and Singapore (Table 3). In Australia, the major bleed incidence per patient was not statistically different for patients with or without statins, but pravastatin was associated with a significantly higher incidence of major bleeds. In Singapore, the incidence of bleeds was significantly lower for patients on any statin compared with no statin therapy for overall bleeds (0.030 vs. 0.071, *p* = 0.0018) and major bleeds (0.008 vs. 0.023, *p* = 0.0374).

## 4. Discussion

Despite the introduction of the new oral anticoagulants (direct thrombin inhibitors and factor Xa inhibitors), warfarin remains widely prescribed. Australia and Singapore have high rates of prescribing oral anticoagulants for stroke prevention [17] and in the Asia–Pacific region, Australia has the highest adjusted rate of warfarin use [18]. Warfarin is economical and well-characterised but requires ongoing monitoring as many factors can influence response, including drug interactions [19]. Warfarin can potentially interact with around 250 different drugs through pharmacokinetic and/or pharmacodynamic interactions [20]. A full list of interactions can be found in resources, such as the Australian Medicines Handbook [21] but the cardiovascular drugs known to interact with warfarin include amiodarone, fibrates, and statins. Statins and warfarin are amongst the most commonly prescribed prescription medications and may be involved in both pharmacokinetic interactions and an increased risk of adverse effects [22]. However, there have been conflicting reports on the risks associated with this combination, particularly regarding warfarin control and bleed risk. Therefore, the aim of this study was to determine the effect of long-term statin administration on warfarin TTR and bleed events in patients with AF in both Australia and Singapore. This study found that warfarin control, as measured by TTR, was not affected by concurrent long-term statin use. Statin use had no effect on bleeds in Australia, whilst, in contrast, patients on statins had a decreased incidence of bleeds in Singapore compared to patients not using statins.

The majority of patients at both sites were prescribed statins (57% in Australia, 73% in Singapore), with atorvastatin most frequently used in Australia, and simvastatin in Singapore. The Australia results are similar to those reported by Gadzhanova and Roughead [23] who found 54% of elderly Australians treated with warfarin for AF were also prescribed a statin, with atorvastatin being the most frequent, followed by simvastatin and rosuvastatin. Similarly, the higher statin use found in Singapore is consistent with Enas et al. [24] who found that South Asians have higher rates of coronary artery disease due to dyslipidaemia. Pravastatin was the least commonly prescribed statin in Australia and Singapore, which is also consistent with Gadzhanova and Roughead [23] who found that the prescription of pravastatin was decreasing, despite the lower potential for a drug interaction with warfarin. Furthermore, pravastatin use has declined since the introduction of more potent statins with longer half-lives such as rosuvastatin and atorvastatin.

At both sites, the TTR of warfarin did not vary according to statin therapy or according to the individual statin prescribed. Similar to this, Shin et al. [13] reported no significant difference in warfarin TTR among patients on four different statins. This is in contrast to Verhovsek et al. [25] who found that patients receiving interacting medicines such as simvastatin spent less time in therapeutic range. Other studies generally report the effect on INR rather than TTR. The studies show conflicting effects. Jindal et al. [26] reported no significant alteration on the effect of warfarin by rosuvastatin, whilst Yu et al. [27] reported that INR increased significantly with rosuvastatin 40 mg. Interestingly, van Rein et al. [28] found that immediate INR increases on statin initiation were insignificant and that the stratification of both INR and dose changes were similar between the different statins. However, this was not with warfarin, but other coumarin derivatives. Coumarin derivatives such as acenocoumarol or fenprocoumon are prescribed in some countries, however, in both Australia and Singapore, warfarin is the only coumarin derivative used. Further to this, patients in this study in both Australia and Singapore had INR tests by the respective clinics and dose adjustments by physicians, but did not utilise self-control methods, including point-of-care testing, which has the potential to influence the frequency of dose changes. Our findings in this study were that the average weekly warfarin dose and the number of warfarin dose changes were not significantly different in patients with or without statins. Previously, both Andrus [29] and Herman et al. [30] found that the limitation of described interactions with warfarin and statins was that the majority of data were only case reports. Further to this, Andrus [29] suggested that the interpatient variability in CYP enzyme activity may explain discrepancies in reports of warfarin and statins and that clinicians should monitor INR closely after starting statin therapy. Our study investigated long-term control and found no effect on warfarin control by statin therapy; however, further investigations may be required regarding the effects on warfarin when statin therapy is either commenced or ceased.

In Australia, bleed events showed no differences according to statin therapy. Similar to this, Leonard et al. [31] found that there was no association with increased serious bleeds when warfarin and statins were used concomitantly in a predominantly Caucasian population. Further to this, in a Canadian population, Douketis et al. [14] associated long-term (≥1 year) statin use with a decreased risk of bleeding on warfarin, but found no association between bleeding and recent statin use (≤6 months). Comparable to this, our study duration was six months and there were no increased bleeds with statins in Australia. Additionally, similar to our study, Suh et al. [32] found no significant change to bleed risk with warfarin and lipid-lowering agents, including statins taken on a long-term basis.

Hori et al. [33] reported rates of total bleeding to be significantly higher in Asian patients compared to non-Asians. Barta et al. [34] found TTR to be a predictor of clinically relevant bleed events, and further to this, Rouaud et al. [35] associated haemorrhagic events with a low quality of warfarin control. In our study, although not reaching significance, the incidence of major bleeds was higher in Singapore than in Australia. The lower TTR in Singapore compared to Australia may explain this higher incidence of major bleeds. Consistent with Chen et al. [17], there was suboptimal control in the Asian populations compared to Caucasian populations. Genetic polymorphisms and differences in concomitant diseases may have been a contributing factor. However, in our study, despite differences in the frequency of co-morbidities, the median HASBLED scores were the same at both sites. However, at both sites, the HASBLED scores were lower in the patients taking no statins. Interesting, only patients in Singapore that were on statins had a statistically lower bleed incidence than patients not on statins. This is similar to the previously mentioned findings by Douketis et al. [14], associating warfarin and long-term statins with decreased bleeding. In an Asian population, Nadatani et al. [36] demonstrated that the use of statins concurrently with aspirin had a tendency to decrease gastrointestinal bleeding. Although not specific to Asian populations, several authors [37,38,39] have previously found that patients receiving statins were less likely to have bleed events when used in combination with antiplatelet agents. Numerous co-prescribed medicines may affect the risk of bleeding with warfarin, including anti-platelets, non-steroidal inflammatory drugs, selective serotonin receptor inhibitors, and proton pump inhibitors [40]. In this study, the bleed incidence and TTR may have been influenced by other potentially interacting medication. Consequently, any future studies assessing the outcomes with warfarin and statins should adjust for all other potentially interacting medications.

Atar et al. [41] found that statins may exert a protective effect against gastrointestinal bleeding in patients with acute coronary syndrome, with the proposed mechanism being that statins increase cyclooxygenase expression and prostaglandin release, with subsequent protective effects on gastrointestinal mucosa. Our study investigated the overall bleed incidences and not specifically gastrointestinal bleeds. Additional investigation is required into the specific locations of bleeding both with and without statins. It also remains unclear why the lowered bleed risk with statins was only in the Asian population. The genetic variants of CYP2C9 can directly increase the risk of warfarin bleeding [42] and variants are more common in Caucasians than in Asians [43]. Further investigation into the particular influence of pharmacogenetics on the outcomes of warfarin and statins with regard to the risk of bleeding may be beneficial in explaining the differences seen in the Asian population. Shin et al. [13] assessed gastrointestinal bleeding among different statins in an Asian population and assessed the associated bleed risk in individual agents. They found that rosuvastatin increased the risk most, followed by atorvastatin, simvastatin, and pravastatin. In Singapore, the overall bleed incidence was not statistically different across the individual statins. Both Andrus [29] and Guidoni et al. [44] found simvastatin to cause an increased risk of bleeding. Further to this, Schelleman et al. [12] found simvastatin, atorvastatin, and fluvastatin were associated with an increased bleed risk, while pravastatin was not. In contrast to this study, in Australia, pravastatin had a statistically higher incidence of major bleeds, compared to the other statins, although patient numbers in this sub-group were relatively low. The retrospective nature of this study may have influenced the reported number of bleeds and limited access to more specific details surrounding bleed events, specifically data on influencing factors and INR at the time of the bleed. In addition, this study included self-reported bleed events. Differences in the frequency of testing at the two sites may have influenced patient recall.

## 5. Conclusions

This study, regarding the influence of long-term statins on warfarin control, found that there was no effect on warfarin control in either Australia or Singapore, as measured by TTR, the testing frequency, the warfarin dose and warfarin dose changes. Statin use did not affect bleed risk in Australia, whilst the bleed incidence was lower in Singapore for patients on statins compared to no statins. The chronic concurrent administration of warfarin and statins is unlikely to affect warfarin control, however, further investigation is still required, especially regarding the lowered bleed incidence in the Asian population and the potential genetic influences on both statin and warfarin metabolism and outcomes.

## Figures and Tables

**Table 1:** Patient demographics at the study sites in Australia and Singapore. Data shown are the number and percentage of patients concurrently prescribed statins or no statins. Mean and standard deviation is also shown for age, and HASBLED (Hypertension, Abnormal renal/liver function, Stroke, Bleeding history, Labile International Normalised Ratio or INR, Elderly > 65 years, Drugs/alcohol concomitantly) score. Statistics shown are for patients on statin therapy in Australia compared to Singapore, and for patients on no statin therapy in Australia compared to Singapore with *** *p* < 0.001 and **** *p* < 0.0001.

	Australia		Singapore	
	Statin (*n* = 1831)	No Statin (*n* = 1365)	Statin (*n* = 859)	No Statin (*n* = 311)
Male	1016 (55.5%)	655 (48.0%)	527 (61.4%)	179 (57.6%)
Female	815 (44.5%)	710 (52.0%)	332 (38.6%)	132 (42.4%)
Age-mean(SD) ****	76.7 (8.6)	77.8 (9.7)	70.1 (9.6)	68.6 (11.0)
Past Medical History				
Hypertension ****	724 (39.5%)	460 (33.7%)	540 (62.9%)	157 (50.5%)
Diabetes ****	411 (22.4%)	151 (11.1%)	274 (31.9%)	77 (24.8%)
Heart Failure ****	157 (8.6%)	121 (8.9%)	70 (8.1%)	18 (5.8%)
Vascular Disease	274 (15.0%)	83 (6.1%)	239 (27.8%)	39 (12.5%)
Chronic Kidney Disease ****	94 (5.2%)	55 (4.0%)	120 (14.0%)	43 (13.8%)
Abnormal liver function	6 (0.3%)	5 (0.4%)	4 (0.5%)	3 (0.9%)
History of stroke or TIA ****	318 (17.4%)	169 (12.4%)	36 (4.2%)	10 (3.2%)
History of bleeds	7 (0.4%)	11 (0.8%)	0 (0%)	0 (0%)
HASBLED score ***	1.7 (0.8)	1.5 (0.8)	1.4 (0.8)	1.1 (0.8)
Concurrent Medication				
Amiodarone ***	149 (8.1%)	66 (4.8%)	59 (6.9%)	17 (1.5%)
Beta Blocker ****	1203 (65.7%)	792 (58.0%)	695 (46.0%)	217 (18.5%)
Digoxin ***	565 (30.9%)	516 (37.8%)	238 (27.7%)	93 (7.9%)
Angiotensin Converting Enzyme Inhibitor ****	695 (38.0%)	421 (30.8%)	272 (31.7%)	67 (5.7%)
Angiotensin Receptor Blocker ***	538 (29.4%)	349 (25.6%)	249 (29.0%)	71 (6.1%)
Calcium Channel Blocker ***	521 (28.5%)	302 (22.1%)	309 (36.0%)	77 (6.6%)
Platelet Inhibitor ****	220 (12.2%)	67 (4.9%)	269 (31.3%)	32 (2.7%)

**Table 2:** Time in therapeutic range (TTR) of patients at the two study sites, according to statin therapy. Numbers and percentages of patients are shown for patients with and without statin therapy, and for each individual statin. The mean (standard deviation) is shown for TTR, testing frequency, and warfarin dose information. The statistics shown are for patients in Australia compared to Singapore in each subgroup with **** *p* < 0.0001.

	Patients	TTR	Testing Frequency	Weekly Warfarin Dose	Warfarin Dose Changes
AUSTRALIA					
Entire Cohort	3196 (100%)	82.4 (15.6)	16.9 (8.1)	26.5 (18.7)	2.5 (2.7)
NO statin	1365 (42.7%)	82.1 (16.1)	17.1 (8.4)	26.8 (25.1)	2.5 (2.8)
Any Statin	1831 (57.3%)	82.5 (15.2)	16.7 (7.9)	26.2 (12.0)	2.5 (2.7)
Atorvastatin	977 (53.4%)	82.0 (15.4)	16.2 (7.5)	26.6 (12.3)	2.7 (2.9)
Simvastatin	354 (19.3%)	83.9 (14.9)	17.4 (8.4)	24.7 (11.0)	2.3 (2.5)
Pravastatin	106 (5.8%)	83.1 (14.7)	17.2 (8.9)	25.6 (12.1)	2.3 (2.7)
Rosuvastatin	392 (21.4%)	82.8 (15.0)	17.3 (8.0)	26.7 (12.1)	2.3 (2.6)
Lovastatin	2 (0.1%)	95.0 (7.1)	22.1 (17.5)	13.7 (4.5)	1 (1.4)
SINGAPORE					
Entire Cohort ****	1170 (100%)	57.6 (34.2)	29.3 (15.2)	18.4 (8.3)	1.0 (1.5)
NO statin ****	311 (26.6%)	59.2 (35.2)	29.7 (15.8)	18.4 (7.9)	1.0 (1.4)
Any Statin ****	859 (73.4%)	57.1 (33.8)	29.1 (15.0)	18.4 (8.4)	1.5 (1.5)
Atorvastatin ****	188 (21.9%)	52.0 (34.0)	27.1 (14.6)	20.6 (10.2)	1.4 (1.4)
Simvastatin****	588 (68.5%)	59.1 (33.6)	29.6 (14.7)	17.8 (7.6)	1.0 (1.5)
Pravastatin ****	5 (0.5%)	78.7 (36.2)	28.5 (14.9)	20.5 (18.3)	0.6 (0.9)
Rosuvastatin ****	31 (3.6%)	53.1 (32.2)	33.9 (19.1)	19.3 (8.7)	1.2 (1.4)
Lovastatin ****	47 (5.5%)	52.8 (34.4)	27.9 (16.1)	16.3 (7.0)	1.1 (1.5)

**Table 3:** The number of events at the two study sites. Data shown are the number of events and incidence per patient for bleeds with and without statin therapy, and for each individual statin. The statistics shown are * overall bleed incidence for entire cohort compared to patients on statins in Singapore with *p* < 0.05, ** overall bleed incidence for patients on no statin compared to statin therapy in Singapore with *p* < 0.01, ^#^ major bleed incidence for patients on no statin compared to statin therapy in Singapore with *p* < 0.05, and ^##^ major bleed incidence for patients on pravastatin compared to each subgroup in Australia with *p* < 0.01.

	Patients	All Bleed Events	Incidence per Patient	Major Bleed Events	Incidence per Patient
Australia					
Entire Cohort	3196 (100%)	138	0.043	25	0.008
NO statin	1365 (42.7%)	54	0.040	10	0.007
Any Statin	1831 (57.3%)	84	0.046	15	0.008
Atorvastatin	977 (53.4%)	47	0.048	7	0.007
Simvastatin	354 (19.3%)	20	0.056	2	0.006
Pravastatin	106 (5.8%)	5	0.047	4	0.038 ^##^
Rosuvastatin	392 (21.4%)	12	0.031	2	0.005
Lovastatin	2 (0.1%)	0	0	0	0
SINGAPORE					
Entire Cohort	1170 (100%)	48	0.041	14	0.012
NO statin	311 (26.6%)	22	0.071 *	7	0.023 ^#^
Any Statin	859 (73.4%)	26	0.030 **	7	0.008
Atorvastatin	188 (21.9%)	5	0.027	2	0.011
Simvastatin	588 (68.5%)	20	0.034	5	0.009
Pravastatin	5 (0.5%)	0	0	0	0
Rosuvastatin	31 (3.6%)	0	0	0	0
Lovastatin	47 (5.5%)	1	0.021	0	0
